# Supplementary material for: Nuclear-Localized Fluorescent Proteins Enable Visualization of Nuclear Behavior in the Basidiomycete Schizophyllum commune Early Mating Interactions
Source: J Fungi (Basel). 2023 Oct 24;9(11):1043. doi: 10.3390/jof9111043 (PMC10671879; doi:10.3390/jof9111043)
Supplement: Supplementary file 1 [file jof-09-01043-s001.zip › jof-2634386-figure S1.pdf]

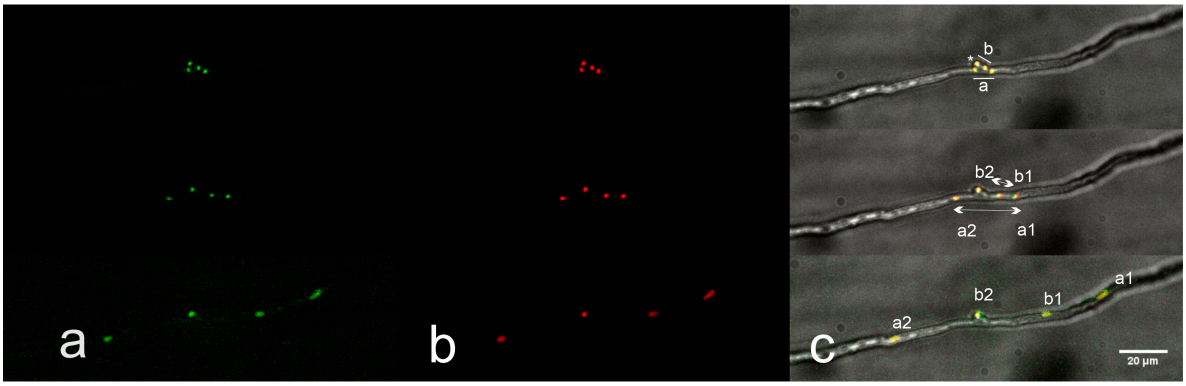

Figure S1. Montage of stacks of a conjugate division. **a**. H2B::EGFP, **b**. H2B::mCherry and **c**. bright field images of the dividing nuclei. The three top images show the synchronous anaphase of the two nuclei (arrows beneath the nuclei) with different mating types, one nucleus (a) divides in the hypha and the other (b) at the base of the developing clamp cell (star). The middle image presents the early and the bottom late telophase. One of the two nuclei from division in hypha moves forwards towards the hyphal tip at the right and the other backwards to the becoming subapical cell. From the division at the base of the developing clamp one nucleus moves towards the hyphal tip and the other remains enclosed in the clamp cell. The images demonstrate that each nucleus contains both green and red labels. The bottom image of **c** is also seen in Figure 1d and in Supplementary material Video S1.
